# Utilising the Intel RealSense Camera for Measuring Health Outcomes in Clinical Research

**DOI:** 10.1007/s10916-018-0905-x

**Published:** 2018-02-05

**Authors:** Francesco Luke Siena, Bill Byrom, Paul Watts, Philip Breedon

**Affiliations:** 1 Medical Design Research Group, Nottingham Trent University, Nottingham, NG1 4FQ UK; 2 Product Innovation, ICON Clinical Research, Marlow, SL7 1YL UK

**Keywords:** 3D Depth Camera, Intel® RealSense™, Motion Capture, Clinical Trials, Health Outcomes

## Abstract

Applications utilising 3D Camera technologies for the measurement of health outcomes in the health and wellness sector continues to expand. The Intel® RealSense™ is one of the leading 3D depth sensing cameras currently available on the market and aligns itself for use in many applications, including robotics, automation, and medical systems. One of the most prominent areas is the production of interactive solutions for rehabilitation which includes gait analysis and facial tracking. Advancements in depth camera technology has resulted in a noticeable increase in the integration of these technologies into portable platforms, suggesting significant future potential for pervasive in-clinic and field based health assessment solutions. This paper reviews the Intel RealSense technology’s technical capabilities and discusses its application to clinical research and includes examples where the Intel RealSense camera range has been used for the measurement of health outcomes. This review supports the use of the technology to develop robust, objective movement and mobility-based endpoints to enable accurate tracking of the effects of treatment interventions in clinical trials.

## Introduction

There are an increasing number of 3D camera-based technologies in today’s market generating a large number of technological solutions in many different sectors. Finding a unique application is therefore becoming even more difficult whether it be related to 3D scanning, motion capture, gaming, wellbeing applications or clinical and healthcare research. The rapid development of 3D camera technologies has resulted in a market with numerous options, all with varying strengths and weaknesses. Therefore, camera selection is critical dependant on the desired application. Some of the most notable solutions include the Microsoft® Kinect™^1^ Sensor V1/1.0 (360), Microsoft Kinect Sensor V2/2.0 (One) (Microsoft Corp., Redmond, Washington, USA), Intel RealSense 3D Camera range, Intel® Euclid™ (Intel Corp., Santa Clara, CA, USA), Creative Senz3D (Creative Technology Ltd., Singapore) Asus Xtion Pro Live (AsusTek Computer Inc., Beitou District, Taipei, Taiwan), OptiTrack MoCap (NaturalPoint, Inc., USA), Vicon Infineon 3D Image Sensor (REAL3™ Gesture Control) (Vicon Motion Systems Ltd., Oxford, UK) and ZED Stereo Camera (Stereolabs Inc. San Francisco, CA, USA).

The Intel RealSense is one of the most prominent 3D depth sensing cameras available, and especially now, considering the recent announcement that manufacture of the Microsoft Kinect Sensor V2/2.0 has now ceased - potentially signalling the end of Microsoft’s involvement in this technology sector [1]. Although Kinect support will continue to exist in the interim for the motion-sensing device, the limited access to purchase this technology will likely signal a change in the adoption and use of this motion sensing device for new development applications.

### Application to clinical research

There are numerous applications utilising motion capture technology to study or encourage movement in wellness, healthcare and clinical research. Much of this work has been accomplished in the area of rehabilitation to provide an engaging environment through which to conduct a regular exercise regimen. This enables patient feedback and correction to ensure that exercises are being performed correctly for optimal benefit, and enables remote assessment and adjustment of exercise regimens between clinic visits [2].

In clinical research and clinical drug development, the study of movement and mobility can be a vital component of understanding the impact of an intervention. This is typically assessed by clinician rating or using an instrumented solution to provide an objective measurement. Many instrumented solutions to assess motion and mobility, such as the Vicon 3D motion analysis system (Vicon Motion Systems Ltd., Oxford, UK) or the GAITRite™ pressure pad system (CIR systems Inc., Franklin, NJ, USA) are expensive to use and may be confined to specialist assessment centres, limiting their use in large-scale clinical trials.

Low-cost, reliable 3D camera technology may provide a means to collect rich objective data in non-specialist centres in large clinical trials. Application areas may include: assessment of gait and upper extremity movement (e.g., in stroke patients); walking, upper extremity and dexterity tests (e.g., in multiple sclerosis); walking, posture and balance testing (e.g., in Parkinson’s disease); and other indications or side-effects where objective measures of movement or mobility are important to how patient’s function such as: amyotrophic lateral sclerosis, tardive dyskinesia, Huntington disease, depression (where gait is indicative of mood status), restless leg syndrome, Bell’s (facial) palsy, muscular dystrophies and rheumatoid diseases amongst many others.

## Properties of the Intel RealSense camera relevant to clinical research applications

The Intel® RealSense™ camera utilises a variety of sensing technologies to achieve depth perception, 3D imaging, interior mapping, and feature tracking. Intel present a variety of uses for the Intel® RealSense™, which considers virtual reality, robotic vision development, drones, security, 3D scanning and tracking, amongst others. Intel have developed a range of camera systems which can be integrated into a variety of platforms including PC’s, laptops, 2-1 Laptop/PC’s, external camera systems, smartphones and tablets.

A number of the capabilities of these camera systems, and those produced by other manufacturers are particularly important to consider when creating applications for clinical research; a selection of these are described in Table 1.

**Table 1:** 3D camera system properties for consideration when developing clinical research applications

Property	Rationale
Field of vision	Field and depth of vision define the working area in which the tracking of patient movement can be achieved. The available working area will define the nature of performance tests that can be developed and measured using the camera system. For example, simple gait and walking tests will require sufficient depth / field of vision to ensure that at least a full gait cycle can be captured for a walking subject not attached to a treadmill.
Depth of vision	
Sample rate	Sample rate is an important consideration to ensure specific movements can be captured to the required level of granularity and precision. Simple performance tests such as measuring shoulder range of motion may not require high sample rates when tests are conducted slowly and only the final range of motion angle is required. However, tests in which rapid movements are conducted, such as the evaluation of movement from toe off to heel strike during walking will require higher sampling rates for accurate estimation.
Resolution	Higher resolution is important in the tracking and measurement of more detailed movement, such as facial analysis or hand joint movements.
Skeletal tracking	Accurate and reliable tracking of 3D joint coordinates is the basis for many rehabilitation and clinical research applications requiring the measurement of body movements and balance.
Facial tracking	Accurate 3D tracking of facial landmarks enables the tracking and measurement of a number of aspects of facial movement such as facial paralysis recovery after stroke / Bell’s palsy and assessment of stimulus-related expression in ADHD and autism spectrum disorders.
Hand and digit tracking	Tracking of hand and digit movement is important in performance tests of dexterity and potentially in the detection of gross tremor movements in conditions such as Parkinson’s disease.
Object recognition	Object recognition is an important component of accurate landmark tracking where estimates may be affected by the presence of additional objects within the field of vision. Object detection is used extensively with face detection and recognition (e.g., detection of glasses, piercings or facial hair), and in skeletal tracking (e.g., presence of a chair or walking support).

All of the 3D camera’s that utilise Intel's RealSense software development kits (SDKs) enable the development of natural, immersive, and intuitive software applications utilising routines that include but that are not limted to, facial recognition, hand gesture, background removal, 3D scanning and skeletal tracking. Another recent addition to the market is the Intel Euclid™ Development Kit. This utilises an Ubuntu® (Canonical Ltd., London, UK) operating system integrated with Intel RealSense depth camera technology in combination with an Intel® Atom™ ×7-Z8700 Quad Core CPU providing a compact and sleek all-in-one computer and depth camera system [3].

### Facial tracking

The Intel RealSense camera range can be used to scan and track facial features and gestures with and without facial hair and glasses. It has the ability to 3D track up to 78 facial landmark points that can support avatar creation, emotion recognition and facial animation [4]. The Intel RealSense can also detect head orientation along 3D axes for yaw, pitch and roll. Depending on the camera in use the ability to track up to four faces with marked rectangles for face boundaries is achievable. For systems where the environment cannot be controlled this is extremely useful as it would allow the individual patient to be identified and solely tracked. The Intel RealSense camera range can offer greater resolution and sampling rates compared to some of the current market leaders, including the Microsoft Kinect 2.0. The greater resolution and sampling rate offer advantages when tracking fine or fast movements.

### Skeletal tracking

The Intel RealSense can also perform skeletal tracking. By tracking the 3D coordinates of body joints, the Intel RealSense can create a virtual skeleton allowing the calculation of patient movement parameters and joint angles. This real-time tracking enables the development of applications in a number of key areas. Tracking a user’s skeletal movement during a routine exercise or while playing a game opens up the ability to detect subtle improvements during rehabilitation from injury or stroke. In the past this has been done by placing sensors on the user’s body at key locations, or using complex multi-camera systems, and the transmitting data to a connected machine for analysis. These setups are often expensive, inconvenient and difficult to use in non-specialist settings. In addition, joint tracking can enable the measurement of body movement attributes such as gait parameters, body sway and balance while the patient is conducting simple performance tests in front of the camera. The ability to conduct these tests simply in small spaces and without attaching sensors or instruments to the patient increases the utility of this approach.

### Intel RealSense camera SR300, R200 & F200

The Intel RealSense SR300 is a short range, coded light 3D imaging system and is one of the smallest 3D depth and 2D camera modules currently available on the market. The Intel RealSense SR300 combines depth sensing with a 1080p RGB camera, this provides users the opportunity to work with dynamic background segmentation, 3D scanning, facial recognition and hand gesture recognition. The Intel RealSense SR300 camera is ideal for face analytics and tracking, scanning and mapping, scene segmentation, hand and finger tracking and augmented reality. This is completed through the utilisation of an infrared (IR) projector and IR camera in tandem using coded light patterns [5].

The Intel RealSense Camera R200 is a USB 3.0 world-facing camera system that can provide colour, depth, and infrared video streams which can be utilised alongside various supported systems such as an Ultrabook™, 2-in-1, All-in-One, or mobile platforms (i.e. smart phones and tablets) [6]. The R200 has a full HD colour camera and IR depth sensing features. Its three cameras provide RGB (colour) and stereoscopic IR to produce depth. The R200 consists of an infrared laser projection system, two infrared and full HD colour imaging sensors. The depth video stream is generated using stereo vision technology which is assisted by the infrared laser projector and the two infrared imaging sensors [7]. The R200 system provides the capability to manipulate filters through varied options including parallax, dolly zoom, motion affects, object segmentation, colour popping, and others [8]. Digitally capturing people or objects in a 3D world by utilising body scans provides the developer with the option to build 3D printable object and components, including the ability to create and manipulate personalised avatars. The creation of 3D objects or avatars provides objects that can be incorporated into real-world applications and spaces which can aid visualisations [8]. The use of avatars has often been used in rehabilitation systems where patients may not like to see themselves after suffering a temporary or permanent physical disability.

The Intel RealSense range has progressively improved over recent years, and this is noticeable especially in the quality of motion capture, camera capabilities and field of view ranges. A full comparison of the general features of the SR300, R200 and F200 systems can be found in Table 2.

**Table 2:** Intel RealSense F200, R200 & SR300 Generic Feature Comparison [9]

	Intel RealSense F200	Intel RealSense R200	Intel RealSense SR300
RGB Camera (Pixel)	1080p at 30 FPS	1080p at 30 FPS	1080p at 30 FPS, 720p at 60 FPS
Depth Camera (Pixel)	Up to 640 × 480 at 60 FPS (Fast VGA, VGA), HVGA at 110 FPS	640 × 480 resolution at 60 FPS	Up to 640 × 480 at 60 FPS (Fast VGA, VGA), HVGA at 110 FPS
RGB Colour Field Of View	43^o^,70^o^,77^o^	77°×43°×70°	41.5^o^,68^o^,75.2^o^
Infrared Field Of View	59^o^,73^o^,90^o^	70°×46°×59°	55°×71.5°×88°
Approx. price (USD)*	140	180	110
SDK Status	Discontinued	Discontinued	SDK 2.0 Capable & Support Active (GitHub)
3D Camera Features			
Effective Range	0.2 m – 1.2 m	0.4 m to 2.8 m	0.2 m – 1.2 m
Texture Mapping	Yes	Yes	Yes
World Mapping	Yes	Yes	Yes
Person Tracking	No	Yes	Yes

The Intel RealSense SDK, SDK components and depth camera managers for the F200, SR300, and R200 versions are no longer being updated due to the introduction of newer versions of the camera range [10], as described below. A full comparison of the SDK algorithm operating ranges and applications can be found in Table 3.

**Table 3:** Intel RealSense F200, R200 & SR300 Camera Algorithm Operating Ranges [11]

	Intel RealSense F200	Intel RealSense R200	Intel RealSense SR300
Facial Tracking			
Detection	30-100 cm	55-250 cm	30-100 cm
Landmark	30-100 cm	50-150 cm	30-100 cm
Recognition	30-80 cm	30-150 cm	30-150 cm
Expression	30-100 cm	30-100 cm	30-100 cm
Pulse	30-60 cm	30-70 cm	30-60 cm
Pose	30-100 cm	50-150 cm	30-100 cm
Hand Tracking			
Hand Segmentation	20-80 (1 m/s)	NA	20-110 (1.5 m/s)
Hand Tracking	20-60 (0.75 m/s)	NA	20-85 (1 m/s)
Hand Gesture Tracking	20-60 (0.75 m/s)	NA	20-85 (1 m/s)
Generic Tracking Features			
Object Tracking	30-180 cm	No	30-180 cm
Person Detection	No	70-350 cm	50-250 cm
Person Tracking	No	70-500 cm	50-500 cm
Skeleton Tracking	No	100-250 cm	50-200 cm
Skeletal Joint Tracking *	No	Yes	Yes

* Skeletal joint tracking no longer supported by Intel RealSense SDKs

The 3D facial tracking capabilities of the Intel RealSense SDK have some important properties that assist with the development of robust and reliable applications. These relate to the ability to provide robust measurements across a range skin tones, and in the presence of additional objects such as piercings or glasses. Facial hair is another factor which can drastically alter the tracking capabilities, however the Intel RealSense SDK can combat this. Obstructions can also greatly affect the tracking capabilities of numerous camera tracking technologies; common movements such as yawning, scratching of the face etc., can affect tracking and data acquisition; the Intel RealSense camera range can also take this into consideration and continue to accurately track.

Variable lighting conditions can adversely affect the quality of the tracking and has been noted in the past to seriously affect the tracking of facial landmarks; this has been especially noted with the Microsoft Kinect [12]. Vision based tracking methods often perform better in controlled environments with limited variables such as skin tone, clothing colour and shape, lighting level, position and restricted background clutter. Utilising this technology in the home for health measurement can be problematic for remote users where the environment may not be ideal [12]. The Intel RealSense, however performs well in low light environments where other solutions continue to struggle but with huge variances in light intensity still being problematic.

**Fig. 1:**
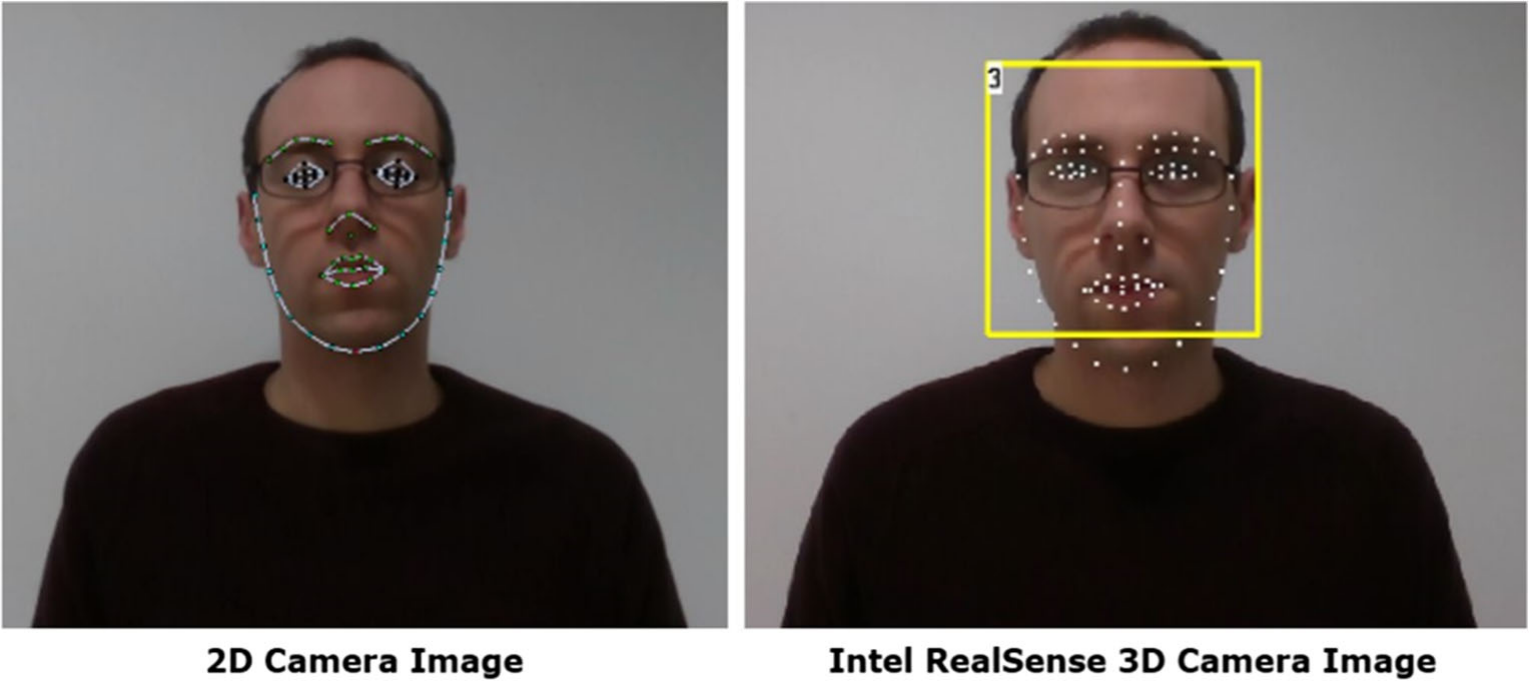
2D (RGB Camera) v 3D (Intel SR300 Camera) Tracking – Glasses

Wearing glasses, in some situations can have an adverse effect on the tracking of the face. This is especially noted with 2D tracking where glasses can significantly affect the size of the eyes which often become amplified by the lenses, thus providing false tracking data (Fig. 1). In the presence of glasses, 2D images may result in loss of landmark tracking, as shown by the black tracking points on the 2D camera image in Fig. 1. This affects the ability to measure and detect actions such as blinking and winking. The use of 3D tracking utilising the SR300 camera in this example is unaffected by the lenses and the frames of the glasses; the tracking points clearly plot the position of the eyes which adjusts based on whether the eyes are open or closed.

**Fig. 2:**
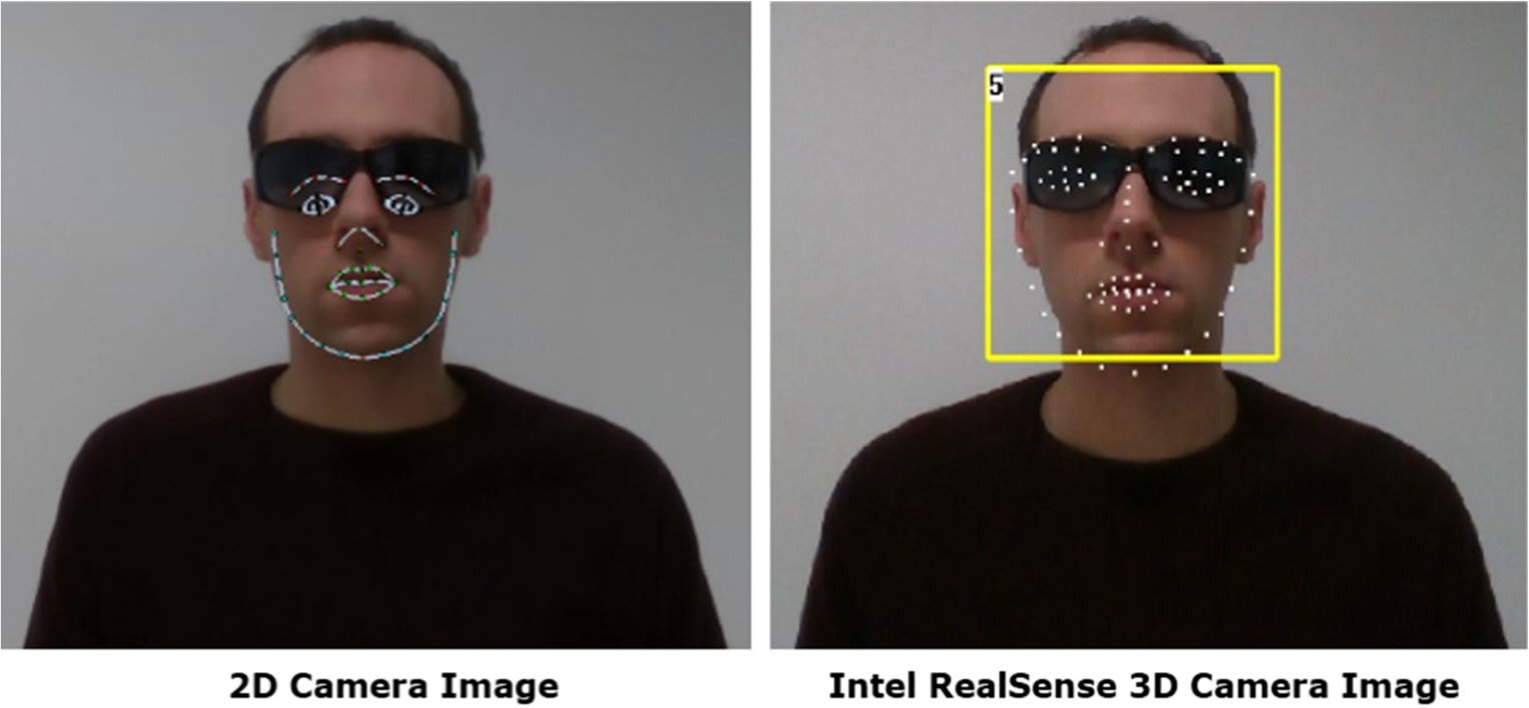
2D (RGB Camera) v 3D (Intel SR300 Camera) Tracking – Tinted Glasses

Variance in lighting and colour can also have an effect on facial tracking. A good example of this is when a subject wears tinted glasses. When utilising 2D tracking the camera is no longer able to track the subject’s eyes and tries to predict where the eyes would be located as a result of the other landmark points. Yet when using 3D camera tracking, the system is able to calculate the location of the eyes accurately thus tracking is unaffected (Fig. 2).

### Intel RealSense camera ZR300, D415 & D435

As the demand for the Intel RealSense and SDK development has advanced, there has become a greater demand for both short and long range depth sensing cameras resulting in the development of the Intel RealSense Cameras ZR300, D415 & D435.

The ZR300 includes a high resolution depth camera which has an integrated robust motion tracking module. The ZR300 has scope for various applications due to its high resolution depth sensing, highly robust tracking, long-range support, indoor and outdoor use, and low power usage. The indoor range of the camera is approximately 0.5-3.5 m with an outdoor range capable of reaching significantly greater distances. However the range supported does vary depending on the light conditions and environment [13]. The Intel RealSense Development Kit ZR300 is seen as an ideal solution for education platforms, hardware prototyping and software development amongst other possible solutions. The ZR300 also has the capability to enable 3D spatial perception using motion tracking, depth perception, and area learning. Most importantly, the ZR300 has a high frame rate which enables tracking performance for 3D scanning applications and rapid motor movements [13]. Table 4 demonstrates that the ZR300 is superior to the SR300; however, the pre-release of the D415 & D435 (Available in Q1 2018) also demonstrates further advancements.

**Table 4:** Intel RealSense SR300, ZR300, D415 & D435 Generic Feature Comparison [14–16]

	IntelRealSense SR300	Intel RealSense ZR300	Intel RealSense D415	IntelRealSense D435
RGB Camera (Pixel)	1080p at 30 FPS, 720p at 60 FPS	2MP, Up to 1080p @ 30 FPS	1920 × 1080 at 30 FPS	1920 × 1080 at 30 FPS
Depth Camera (Pixel)	Up to 640 × 480 at 60 FPS (Fast VGA, VGA), HVGA at 110 FPS	Up to 628 × 468 @ 60 fps	Up to 1280 × 720 at up to 90 FPS	Up to 1280 × 720 at up to 90 FPS
Depth Field of View (FOV)	55°×71.5°×88°	70° × 46° × 59°	69.4°×42.5°×77°	91.2°×65.5°×100.6°
RGB Colour Field Of View	41.5^o^x68^o^x75.2^o^	75° × 41.5° × 68°	69.4°×42.5°×77°	69.4°×42.5°×77°
Effective Depth Range	0.2 m – 1.2 m	0.5 – 3.5 m +	0.3 – 10 m +	0.2 – 10 m +
Typical Environment Of Use	Indoor	Indoor & Outdoor	Indoor & Outdoor	Indoor & Outdoor
Face Tracking & Recognition	Yes	Yes	Yes	Yes
Expression Recognition	Yes	Yes	Yes	Yes
Gesture Recognition	Yes	Yes	Yes	Yes
Hand Tracking	Yes	Yes	Yes	Yes
Approx. price (USD)*	110	289	149	179

* Prices as of November 2017

Intel have recently launched a new D400 camera range that presents the most advanced depth perception capabilities to date. The D415 & D435 depth sensing cameras contain the latest Intel RealSense vision processor and module. The addition of the rolling image shutter and standard field of view, the Intel RealSense Depth Camera D415 offers a general-purpose solution with simple depth stream data capture. However, the D435 offers a global image shutter and wider field of view with the capability to capture and stream the depth data of moving objects thus, providing high depth perception accuracy in motion [16]. In comparison to earlier versions of the Intel RealSense Camera range the optimal environment for the D400 series has been significantly increased with data capture possible at distances up to and in excess of 10 m in both indoor and outdoor environments [16].

## Application to clinical research

Clinical trials employ a variety of approaches to measure the health status of patients and changes in their health due to treatment. In many therapy areas, subjective ratings made by the patient (patient reported outcome measures – PROs) or by a clinician (clinician reported outcome measures – ClinROs) are used to measure status or change over time. Patient reported outcomes measures, such as a daily symptom diary or a quality of life questionnaire importantly measure the perspective of the patient regarding the effects of treatment, and in some cases (e.g. pain assessment) may be the only way to assess efficacy. ClinROs include measures of symptoms through direct observation of the patient. For example, depression symptoms and severity may be rated by a trained clinician following a structured interview using the Hamilton Depression Rating Scale. A further clinical outcome assessment, the performance outcome (PerfO), is a measurement by a healthcare professional based upon observation of a task performed by the patient. In many cases these PerfOs are measured using subjective assessments. Quite a number of subjective measurement scales are used in clinical trials to assess balance, movement or mobility based on observation of the patient conducting a specified movement or activity.

Subjective ratings (e.g. assessing the patient using a verbal response scale) are not very sensitive to detecting small improvements, and different investigators may rate patients differently based upon their interpretation of the scale requirements. For this reason it is sometimes difficult to make measurements that are sensitive enough to detect treatment-related changes and are able to conclusively show treatment effects when they exist. In addition, using investigator observation it is less likely that detailed or subtle aspects of movement and mobility can be recorded. Where these can be replaced by objective measures, this may result in a greater ability to identify changes due to treatment.

**Fig. 3:**
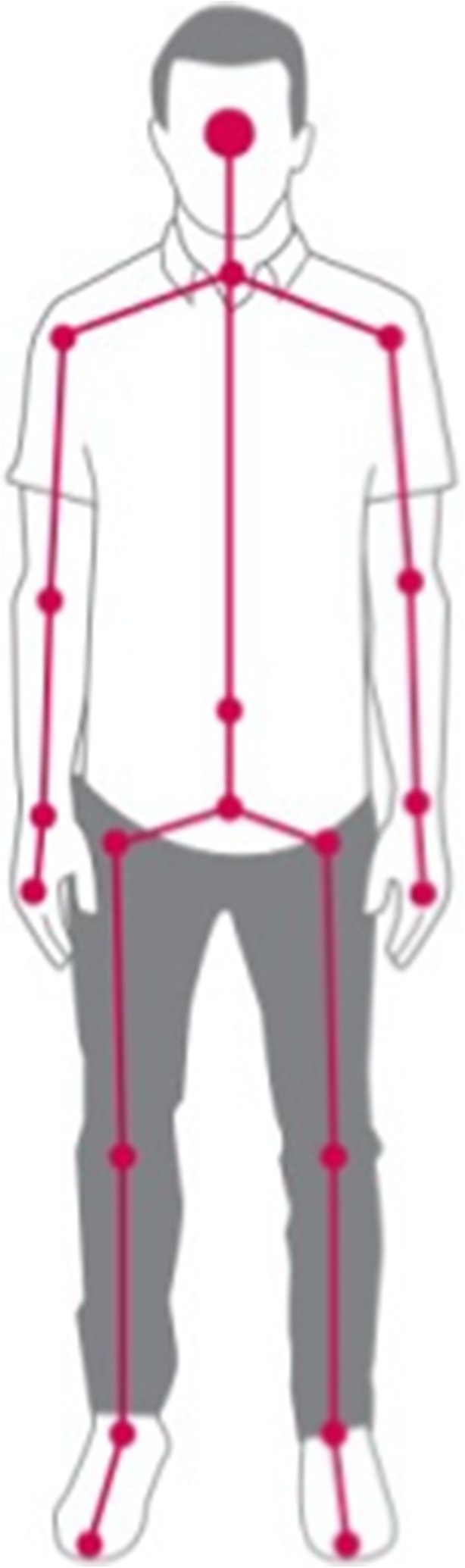
Tracking the 3D position of 26 body joints using Microsoft Kinect

There is a growing body of applications leveraging depth camera platforms to provide applications in healthcare. Much of this work to date has been conducted using Microsoft Kinect and the depth camera utilised within the Microsoft X-box gaming platform. Reasons for this include the low cost and availability of the hardware, the ability to connect to a Windows computer, and the free availability of its SDK which contains skeletal tracking (Fig. 3) and facial analysis modules. The majority of healthcare applications reported using Microsoft Kinect are applied to the area of rehabilitation, including novel ways of engaging patients in regular exercise regimens and solutions to ensure that exercises are performed correctly for optimal outcomes. Published examples apply to a number of disease indications including stroke [17], Parkinson’s disease [18], Multiple Sclerosis [19] and Cerebral Palsy [20].

While many published applications fall short of measuring and reporting specific health outcomes, there are examples of applications built using the Kinect platform that report health outcome measures that may enable the longitudinal tracking of change and the formal assessment of treatment benefits. We provide a good summary of published applications measuring aspects of gait and balance, upper extremity movement, and chest wall motion elsewhere [2, 21].

### Published clinical research applications using the Intel RealSense camera

Less work is available in the published literature on clinical research applications using the Intel RealSense camera range, although this is emerging and likely to increase for the reasons cited earlier in this article. In the absence of a significant body of work, we present some examples below, as opposed to a formal systematic review.

Chhor et al. [22] report the development and preliminary use of a computer game leveraging the Intel RealSense camera to assist upper body rehabilitation and exercising using defined hand gestures and movements. Ferche et al. [23] used the Intel RealSense camera to provide precise tracking of the movements of the human hand within a neuromotor rehabilitation application, and Baldominos et al. [24] and Cidota et al. [25] leveraged the Intel RealSense camera within a rehabilitation system focussed on upper extremity movement.

House et al. [26] utilised the SR300 camera in the evaluation of its use for image-guided interventions and application in vertebral-level localization. Silver et al. [27] also utilised the facial tracking capabilities of an Intel RealSense camera in the production of a happiness and sadness recognition system. Bandini et al. [28] also produced a video-based tracking system utilising the SR300 to capture jaw movements during speech which may be utilised in various health measurement tasks such as the assessment, diagnosis and treatment of various speech disorders.

The Intel RealSense cameras have also been integrated into smart systems which utilise additional hardware. One example in health research utilises the cameras to produce a 3D full-body scan in combination with the Intel® Edison™ module to provide additional peripheral device communication via Bluetooth and Wi-Fi [29].

## Translating outcome data into acceptable trial endpoints

As researchers continue to develop applications using 3D camera technology for use in clinical research, it is important to be cognisant of the requirements associated with the evidence needed to support the use of technology, and the clinical endpoints derived for use, in regulatory clinical trials and new drug applications. In clinical trials, a study endpoint is defined as a characteristic or variable that reflects how a patient feels, functions, or survives [30]. An endpoint description includes information defining how and when they are measured, how they are calculated, rules for missing data and how they are analysed. In the absence of formal regulatory guidance, the Critical Path Institute’s ePRO Consortium reported consensus recommendations on the evidence required to support wearable device selection and endpoints derived from wearables data [31]. These recommendations have strong parallels to the requirements in defining clinical endpoints for regulatory drug submissions arising from outcomes data collected using motion-based cameras.

In essence, it is important to demonstrate the reliability and validity of outcomes data collected using a motion-based platform; and to demonstrate the suitability and interpretability of endpoint measures derived from this data.

### Reliability

Intra- and inter-device reliability should be demonstrated by assessment of test-retest reliability using the same and different units of the same device. Typically this will be assessed using the Intraclass Correlation Coefficient (ICC). To ensure reliability is maintained, camera manufacturers must be able to demonstrate that devices are produced in adherence with a quality system to ensure equivalence of devices between batches and with the reliability data provided. Changes to the SDK affecting the detection of 3D coordinates of body joints will require re-assessment of reliability should software upgrade to leverage the revised SDK.

### Concurrent validity

Concurrent validity is important to demonstrate that the approach is truly measuring what is intended. This is typically performed by comparing results to a gold standard methodology that is regarded as an accurate measure of the concept of interest. For example in the case of body movement, a gold standard could be the VICON camera system. A small study of human subjects should enable this to be evaluated.

### Content validity

It is important to demonstrate that the endpoint(s) derived are considered important to patients and a relevant outcome within the disease/treatment studied. This content validity can be obtained through qualitative data collection in patients or other reporters such as physicians or carers. Some outcomes measures generated by depth camera software may already be well known and understood, and in these cases content validity may be already established.

### Ability to detect change

Outcome measures and derived endpoints should, when used in a clinical trial be seen to be sensitive enough to detect change when a change exists. This is normally demonstrated by controlled studies involving an intervention that is understood to create a change in the outcome of interest.

### Endpoint interpretability

For an endpoint to be suitable for use in a clinical drug submission it is important to understand meaningful change. In other words, it should be understood what degree of change in the endpoint can be interpreted as clinically relevant to the patient. This may be represented by the minimal important difference (MID) or minimally clinically important difference (MCID), or the minimal individual change that distinguishes a responder from a non-responder. There are well established methodologies used to estimate MCID and responder definition [31].

## Discussion and conclusions

The Intel RealSense has considerable potential for research solutions and platforms. Its versatility enables integration into a variety of platforms while remaining a low-cost solution. To date the most popular 3D motion sensing camera technologies utilised within research and development are the Microsoft Kinect 1.0 & 2.0 (X-Box 360 and X-Box One Kinect). The popularity of the Microsoft Kinect 1.0 & 2.0 is not surprising due to the constant development of the SDK’s and gaming applications, this has resulted a discontinuation of a number of 3D camera technologies over the past 10 years. However, the emergence of the Intel RealSense camera range provides an alternative, which the authors believe has comparable and superior technical functionality. The Intel RealSense range is currently in its infancy yet expanding quickly with an ever-improving camera and increasingly extensive SDK. The Intel RealSense system can be seen as a comparable if not superior alternative to the Microsoft Kinect due to its 3D scanning, facial recognition and hand gesture recognition capabilities and therefore this is seen as suitable technology for research and development within the healthcare sector. In addition, with recent announcement that the manufacture of the Microsoft Kinect Sensor V2/2.0 has now ceased [1], potentially signalling the end of Microsoft’s involvement in this technology sector, the Intel RealSense camera range is best placed to take advantage within the clinical and healthcare sector.

The associated SDK is currently the area which limits the full utility of the Intel RealSense camera range. However, as the product is very much in its infancy, this is expected to become more extensive over time. Using the facial tracking abilities of the Intel RealSense, researchers can develop systems that can track a user’s emotions, this can not only be useful for medical products that utilise the camera within the project itself but also for measuring the user acceptability on projects not utilising the camera [32]. Research has also been performed to analyse the feasibility of using skeletal tracking to detect a user’s emotional state based upon their walking gait [33].

The facial and hand tracking abilities of the RealSense can be used to allow a user to interact with the world around them using only small motions. There are many systems utilising the facial and hand tracking capabilities of the Microsoft Kinect such as the Face Controller system for patients with disabilities developed by Risenbit [34]. Patients with limited mobility often require systems to be designed for them to facilitate their disability thus allowing them to perform daily activities such as typing on a keyboard [34]. The best known of these types of systems is the wheelchair used by Professor Stephen Hawking, this has been designed and developed by Intel and currently utilises an IR sensor to detect cheek movements that allow the user to type on a keyboard. Using facial/hand tracking this could be implemented for other users allowing the utilisation of customisable facial or hand gestures and potentially increasing the number of controls available by monitoring multiple areas of the face/hands.

The Intel RealSense camera allows for skeletal hand tracking of 25 tracked body joints, the potential for medical applications range from tracking the full body motion, balance and tracking dexterity and hand movements such as tracking and monitoring a patient with Parkinson’s disease to detect deterioration or improvement in the patient’s condition [35]. Full hand skeletal tracking can be used to track both hands while a patient performs sign language this can then be interpreted and translated for use with typing and voice synthesisers [36]. As depth camera technology becomes more advanced and the components become smaller we are seeing a number of other manufacturers entering the depth camera market and implementing them into mobile phones and tablets. This includes Apple's iPhone X with a depth camera used for facial recognition and tracking. Qualcomm (Qualcomm Incorporated, San Diego, California, USA) have also announced their Spectra™ ISP 2nd generation Module which incorporates high-resolution depth sensing with over 10,000 points of depth for motion tracking. This importantly opens the possibility of developing applications to deliver performance testing remotely and more frequently without the need for patients to visit clinical sites for assessments.

## Footnotes

1 Microsoft ® Corporation, Redmond, Washington, USA, https://www.microsoft.com
